# *osm-5p-* driven fluorophores are differentially expressed in *ccpp-1Δ* and *nekl-4Δ* mutant ciliated neurons

**DOI:** 10.17912/micropub.biology.001245

**Published:** 2024-07-25

**Authors:** Kaiden M Power, Maureen M Barr

**Affiliations:** 1 Department of Genetics and Human Genetics Institute of New Jersey, Rutgers, The State University of New Jersey, New Brunswick, New Jersey, United States

## Abstract

Intraflagellar transport (IFT) involves the coordinated transport of molecular motors and other proteins and is required for ciliogenesis and ciliary maintenance. The *C. elegans* IFT protein OSM-5 /IFT88 is expressed in a majority of the ciliated neurons in the animal, and *osm-5* mutants exhibit structurally defective cilia. The *osm-5* promoter is commonly used to express genetic constructs in the ciliated neurons. In this study, we show that brightness of *osm-5p-* driven constructs is altered in mutants of the tubulin deglutamylase *ccpp-1* and the NIMA-related kinase *nekl-4* . This raises the possibility that *osm-5* expression levels may be regulated by *ccpp-1* and *nekl-4* .

**Figure 1. osm-5p- driven soluble GFP differs in brightness in ccpp-1Δ and nekl-4Δ mutant ciliated neurons:**
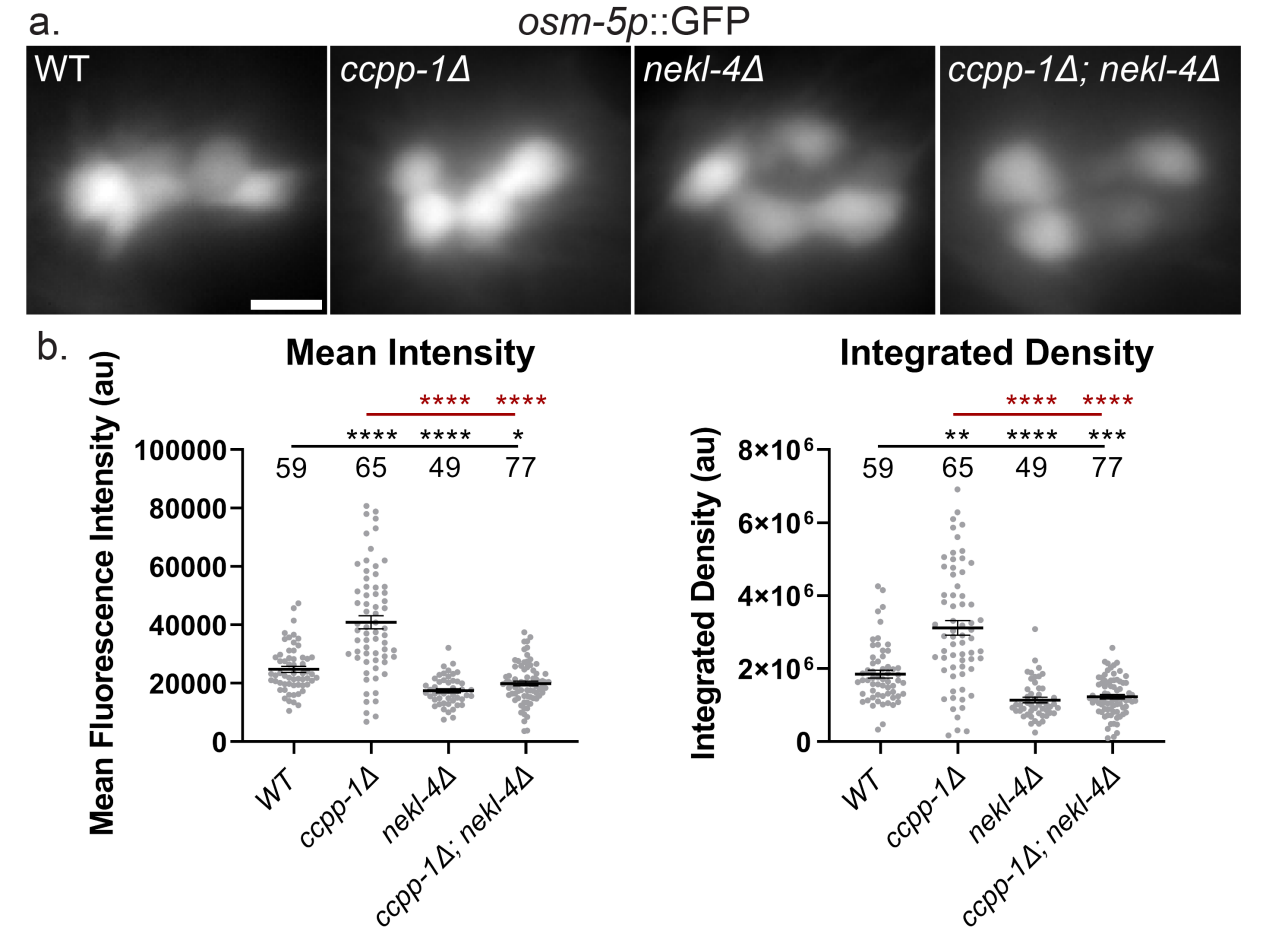
**a.** Widefield images of soluble *osm-5p* ::GFP in the phasmid soma, sum intensity projections. Scale bar = 5 µm. **b.** Quantification of mean fluorescence intensity and integrated density of *osm-5p* ::GFP fluorescence in the phasmid soma. au = arbitrary unit. Mean ± SEM; * indicates p ≤ 0.05, ** indicates p ≤ 0.01, *** indicates p ≤ 0.001, **** indicates p ≤ 0.0001 by Kruskal-Wallis one-way ANOVA with post hoc Dunn's correction for multiple comparisons. n is indicated above each genotype. Black indicates significance relative to WT, red indicates significance relative to *ccpp-1Δ.*

## Description

Cilia are built and maintained by the coordinated transport of molecular motors and other proteins, collectively named intraflagellar transport (IFT) (Reiter and Leroux, 2017) . *C. elegans* OSM-5 is an IFT-B protein homologous to mammalian IFT88, which is required for primary cilium assembly (Pazour et al., 2000; Qin et al., 2001) . Sensory cilia are severely shortened and deformed in *osm-5* mutants, and expectedly, OSM-5 is expressed in a majority of the ciliated neurons in *C. elegans* (Perkins et al., 1986; Haycraft et al., 2001) *.* Due to its pan-ciliary expression and short (240 bp) length, the *osm-5* promoter is commonly used to drive expression of transgenic constructs in the ciliated neurons. In this study, we uncovered differential brightness levels of *osm-5p-* driven constructs that may indicate transcriptional regulation of *osm-5* by the cilia-related proteins CCPP-1 and NEKL-4 .

Previously, we examined localization and brightness of multiple *osm-5p* -driven extrachromosomal constructs to study ciliary and mitochondrial functions of the tubulin deglutamylase CCPP-1 and the Never-In-Mitosis A (NIMA)-related kinase NEKL-4 (Power et al., 2024) . In both *ccpp-1Δ* and *nekl-4Δ* mutants, we observed differences in the brightness of *osm-5p* -driven TOMM-20 ::tagRFP and roGFP. When quantified, total levels of roGFP in the phasmid soma were significantly increased in *ccpp-1Δ* mutants and decreased in *nekl-4Δ* and *ccpp-1Δ; nekl-4Δ* mutants (Power et al., 2024, Figure S5a). To determine if this phenomenon was array-specific, we quantified the fluorescence intensity of soluble *osm-5p* ::GFP in the phasmid soma of *ccpp-1Δ* and *nekl-4Δ* mutants. Sum intensity projections showed a slight increase in brightness in *ccpp-1Δ* mutants, and a slight decrease in *nekl-4Δ* single and *ccpp-1Δ; nekl-4Δ* double mutants ( **Fig 1a** ). When quantified, both the mean fluorescence intensity and integrated density were significantly increased in *ccpp-1Δ* mutants and significantly decreased in *nekl-4Δ* and *ccpp-1Δ; nekl-4Δ* mutants ( **Fig 1b** ). This may indicate that *nekl-4* promotes transcription of *osm-5* while *ccpp-1* represses *osm-5* transcription, and that *nekl-4* is epistatic to *ccpp-1* with respect to *osm-5* expression regulation.

Further studies, including transcriptomics and quantitative PCR, are necessary to definitively determine the effects of *nekl-4* and *ccpp-1* mutation on *osm-5* transcription, as well as the effects of these mutations on the expression of other ciliary genes. It has been shown that expression levels of *nekl-4* are tightly regulated via RNA editing in order to prevent overexpression of NEKL-4 and other kinases to prevent ciliary instability (Li et al., 2021) . Therefore, it is possible that NEKL-4 protein levels influence transcription of *osm-5* and other ciliary genes. The *osm-5* promoter contains an X-box motif, which is found in the promoters of many mammalian and *C. elegans* genes involved in cilia and ciliopathies (Chen et al., 2006) . This presents the possibility that *nekl-4* and *ccpp-1* may affect expression of other cilia-related genes with X-box-containing promoters, such as *dyf-5 , bbs-5 ,* and *che-11* . Further experiments with these mutants would provide valuable insight into the complex regulatory mechanisms that underlie ciliary protein expression.

## Methods

*Widefield imaging*

Live animals were anesthetized with 10 mM levamisole and mounted on 10% agarose pads for imaging at room temperature. Widefield images were acquired on a Zeiss Axio Observer with Colibri 7 LEDs and ZenBlue software (Carl Zeiss Microscopy, Oberkochen, Germany) using a Photometrics Prime 95B sCMOS camera (Teledyne Photometrics, Tucson, AZ). A 63x/1.4 Oil Plan-Apochromat objective was used for imaging. Acquisition settings were identical for all genotypes.

*Quantification of GFP brightness*

Image files were imported into Fiji/ImageJ with the BioFormats Importer plugin and sum intensity projections including the entire set of phasmid soma were created. Images were duplicated and used to create ROIs including only the phasmid soma by thresholding. The mean fluorescence intensity and integrated density within the ROI were measured. Kruskall-Wallis one-way ANOVA analysis and posthoc Dunn's multiple comparison test were performed in Prism (Graphpad Software).

## Reagents

PT2700 : *pha-1 ( e2123 )III; him-5 ( e1490 )V; myEx819 [osm-5p::GFP + pBx]*

PT3826 : *pha-1 ( my82 [ pha-1 ( e2123 )+SnaBI]) nekl-4 ( tm4910 )III; him-5 ( e1490 )V; myEx819 [osm-5p::GFP + pBx]*

PT3837 : *ccpp-1 ( ok1821 )I; pha-1 ( e2123 )III; him-5 ( e1490 )V; myEx819 [osm-5p::GFP + pBx]*

PT3838 : *ccpp-1 ( ok1821 )I; pha-1 ( my82 [ pha-1 ( e2123 )+SnaBI]) nekl-4 ( tm4910 )III; him-5 ( e1490 )V; myEx819 [osm-5p::GFP + pBx]*
